# Integrated gut microbiota and metabolome analysis reveals the mechanism of Xiaoai Jiedu recipe in ameliorating colorectal cancer

**DOI:** 10.3389/fonc.2023.1184786

**Published:** 2023-06-22

**Authors:** Wenli Qiu, Hui Xie, Haibin Chen, Hongli Zhou, Zhongqiu Wang, Hongguang Zhou

**Affiliations:** ^1^ Department of Radiology, Affiliated Hospital of Nanjing University of Chinese Medicine, Nanjing, China; ^2^ The First Clinical Medical College, Henan University of Chinese Medicine, Zhengzhou, China; ^3^ Science and Technology Department, Jiangsu Collaborative Innovation Center of Traditional Chinese Medicine Prevention and Treatment of Tumor, Nanjing University of Chinese Medicine, Nanjing, China; ^4^ College of Pharmacy, Nanjing University of Chinese Medicine, Nanjing, China; ^5^ Department of Oncology, Affiliated Hospital of Nanjing University of Chinese Medicine, Nanjing, China

**Keywords:** traditional Chinese medicine, Xiaoai Jiedu recipe, colorectal cancer, gut microbiota, metabolic profiles

## Abstract

**Introduction:**

Xiaoai Jiedu recipe (XJR), a classical prescription of traditional Chinese medicine (TCM), has been clinically proven to be effective in ameliorating colorectal cancer (CRC). However, its exact mechanism of action is still elusive, limiting its clinical application and promotion to a certain extent. This study aims to evaluate the effect of XJR on CRC and further illustrate mechanism underlying its action.

**Methods:**

We investigated the anti-tumor efficacy of XJR *in vitro* and *vivo* experiments. An integrated 16S rRNA gene sequencing and UPLC-MS based metabolomics approach were performed to explore possible mechanism of XJR anti-CRC on the gut microbiota and serum metabolic profiles. The correlation between altered gut microbiota and disturbed serum metabolites was investigated using Pearson’s correlation analysis.

**Results:**

XJR effectively displayed anti-CRC effect both *in vitro* and *in vivo*. The abundance of aggressive bacteria such as *Bacteroidetes, Bacteroides*, and *Prevotellaceae* decreased, while the levels of beneficial bacteria increased (*Firmicutes*, *Roseburia*, and *Actinobacteria*). Metabolomics analysis identified 12 potential metabolic pathways and 50 serum metabolites with different abundances possibly affected by XJR. Correlation analysis showed that the relative abundance of aggressive bacteria was positively correlated with the levels of *Arachidonic acid*, *Adrenic acid*, *15(S)−HpETE*, *DL−Arginine*, and *Lysopc 18:2*, which was different from the beneficial bacteria.

**Discussion:**

The regulation of gut microbiota and related metabolites may be potential breakthrough point to elucidate the mechanism of XJR in the treatment of the CRC. The strategy employed would provide theoretical basis for clinical application of TCM.

## Introduction

Colorectal cancer (CRC) is currently the second most commonly diagnosed malignancy in China, the incidence of CRC has increased by an average of 550,000 per year due to the complex interactions among genetic, environmental, economic development and lifestyle factors(1). Current the main treatments for CRC are surgery, radiotherapy and chemotherapy, but the therapeutic effect is not optimistic with high rate of recurrence and accompanied obvious side effects(2). Therefore, the prevention and treatment of CRC have attracted worldwide attention in the field of drug research and development, and the more effective, safer, and cheaper anti-tumor drugs need to be developed.

It is well known that cancer is a complex disease, which is generally caused by a combined action of multi-factors in nature (3). Therefore, monotherapy does not necessarily produce ideal efficacy for malignant tumors. With the characteristics of “multi-components” and “multi-targets”, Traditional Chinese Medicine (TCM) has been applied as the principle or auxiliary drugs in the treatment of various complex diseases for more than 2,500 years(4). TCM contributes to promoting homeostasis mainly in two ways: (1) it contains plentiful active ingredients, which usually provide beneficial synergy by acting on diverse biological targets; (2) most of them are natural herbs, sometimes even edible, so they with fewer side effects and low toxicities. Xiaoai Jiedu recipe (XJR) is a classical anti-tumor prescription proposed by Zhou Zhongying, a master of TCM, based on the cancer toxin theory and after his years of clinical practice experience(5). The formula is composed of 7 herbs, *Hedyotis diffusa* (20* g*), *Radix pseudostellariae* (15 g), *Akebia trifoliata Koidz* (12* g*), *Radix ophiopogonis* (12* g*), *Bombyx batryticatus* (10* g*), *Cremastra appendiculata* (10* g*), and *Centipede* (3* g*) in a specific proportion. Although its efficacy in CRC treatment has been confirmed by a number of clinical applications, its exact mechanism of action is still elusive, limiting its clinical application and promotion to a certain extent (6).

The microbiota is an integral part of the body composition, which is equivalent to a virtual organ and plays an important role in maintaining intestinal physiological functions and regulating immunity. As the location with the highest distribution density of microbiota, the imbalance of microbiota can lead to colorectal disorder (7). Recently, accumulating evidences have verified that intestinal flora plays an essential role in the occurrence and development of CRC (8, 9). Chen et al. found that *C. butyricum*, as a kind of butyric-producing bacteria commonly used in clinical treatment of gastrointestinal diseases, could play an anti-intestinal tumor role by modulating the gut microbiota composition, as demonstrated by increases in some beneficial bacteria and decreases in some pathogenic bacteria (10). Flemer et al. found that the microbiota profiles of CRC patients was differ from those of healthy subjects, the abundance of *Bacteroidetes Cluster 1* and *Firmicutes Cluster 1* in the intestinal mucosa of CRC patients decreased, while the abundance of *Bacteroides cluster 2*, *Firmicutes cluster 2*, *Pathogen cluster* and *Prevotella cluster* increased (11). TCM can play an anti-tumor role by regulating the intestinal flora structure (12, 13). In the previous study, we found that Wenzi Jiedu Recipe (WJR) could effectively inhibit the development of CRC by regulating the structure of gut microbiota *via* enriching *Oscillibacter* and *Bacteroides_acidifacien* (14).

Intestinal flora is also the main participant and regulator of host metabolism, so the metabolic process is not only regulated by gene, but also by symbiotic bacteria (15). Therefore, it is vital to evaluate the alterations of intestinal flora-related metabolic phenotype. The combination of microbiological technique and metabolomics provides an effective means for further study of the relationship between host gut microbiota and metabolism, and contributes to in-depth analysis of the molecular mechanism of drug action (16).

In the present study, we confirmed the anti-CRC effect of XJR *in vitro* and *vivo*. 16s rRNA sequencing analysis was conducted to evaluate the effect of XJR on intestinal flora regulation, and UPLC-Q-TOF/MS-based serum metabolomics analysis was performed to find specific metabolic biomarkers and metabolic pathways related to XJR anti-CRC. Moreover, correlation analysis of the specific gut microbiota profiles and serum metabolites was established to understand the potential anti-CRC mechanisms of XJR. We hope the obtained results would provide a reliable scientific basis for promoting the TCM in the clinical treatment of complex diseases.

## Materials and methods

### Preparation of Xiaoai Jiedu recipe

The herbal formula XJR is a combination of seven medicinal herbs: Hedyotis diffusa (20 g), Radix pseudostellariae (15 g), Akebia trifoliata Koidz (12 g), Radix ophiopogonis (12 g), Bombyx batryticatus (10 g), Cremastra appendiculata (10g), and Centipede (3 g). The seven different herbal ingredients that constitute XJR were purchased from Anhui Bozhou Traditional Chinese and Western Medicine Co., Ltd. (Anhui, China) and authenticated by two experienced pharmacists. XJR was decocted according to the standard method and concentrated to 2.0 g/ml, diluted with normal saline (v/v, 1:1), and stored at 4 °C.

### Preparation of medicated serum

18 adult Sprague-Dawley (SD) rats weighing 220~250 g were randomly divided into three groups: control group (NC, normal saline), low-dose group (LD, XJR 5.0 g/kg), and high-dose group (HD, XJR 20.0 g/kg), and each group contained six animals. Rats were gavaged with the different doses of XJR or normal saline twice a day for 3 consecutive days. Within one hour after the last administration, we collected blood samples from rats through retinal vein plexus and obtained the medicated serum through centrifugation and filtration. The XJR-containing serum was stored at –80°C for standby.

### Cell proliferation assays

MTT assay was used to determine cell viability in order to verify the anti-tumor effect of XJR *in vitro*. Briefly, colorectal adenocarcinoma DLD-1 cells were seeded in 96-well plates at 1×10^4^/pore. After incubation for overnight at 37°C in 5% CO_2_ to allow cell attachment, different concentrations of drug serum were added to the wells and incubated for 48 h. After treatments, the cells were further incubated MTT (Sigma St. Louis, MO, USA) for 4 h. Thereafter, 150 µL/well of DMSO (Sigma St. Louis, MO, USA) was added to the plates. The optical density (OD) at a wavelength of 492 nm was measured using a multifunctional microplate reader (Thermo Scientific., Waltham, MA, USA).

### Animal study

Male BALB/c mice (4-6 weeks of age, 18–20 g) were obtained from the Qinglongshan Experimental Animal Center (SCXK-2017-0007) and kept under SPF conditions. The mice were raised in accordance with the guideline for laboratory animals in the Declaration of Helsinki. This animal experiment was approved by the Ethics Committee of Nanjing University of Chinese Medicine (202204A058).

HCT116 cells were subcutaneously administered into the axillary region of the mice to establish the CRC tumor bearing mice model. Based on the conventional dosage of clinical human body and using the dosage conversion formula, we obtained the high (XJR 20.0 g/kg, HD) and low dosage (XJR 5.0 g/kg, LD) for mice(5). Therefore, when the tumors volume reached to 50 mm^3^, the mice were randomly divided into three groups, with six in each group: ① NC group, gavaged with equal volume of normal saline; ② LD group of XJR, gavaged with 5 g/kg XJR; ③ HD group of XJR, gavaged with 20 g/kg XJR. All mice were administrated once a day for 14 days with different concentrations of drug. The tumor volume was calculated as follows: length × width^2^ × 0.5, and tumor growth inhibition rate (TGI) was estimated as follows: (tumor volume in the NC group – tumor volume in the treated group)/tumor volume in the NC group ×100%.

### Histopathology

Xenograft tumors of mice were harvested from each group, preserved in 4% paraformaldehyde, embedded in paraffin, and then cut into 4 μm thick tissue slices as described. After the sample slices were dehydrated with gradient ethanol, they were stained with hematoxylin and eosin (H&E). The sections were assessed by a pathologist using an optical microscope.

### Gut microbiota analysis

After administrating with different agents, we collected the mice feces for gut microbiota analyses(14). The stool DNA was extracted using the E.Z.N.A. soil kit (Omega Bio-tek, Norcross, GA, U.S.) as per manufacturer instructions. The V3–V4 region of bacteria 16S rDNA gene was amplified by PCR (pre-denaturation at 95°C for 3min, followed by 27 cycles at 95°C for 30s, 55°C for 30s, and 72°C for 30s and a last extension at 72°C for 5min) using the primers 338 F (5’-barcode-ACTCCTACGGGAGGCAGCA-3’) and 806R (5’-GGACTACHVGGGTWTCTAAT-3’) to analyze the classification composition of bacterial community. The PCR product was purified with the AxyPrep DNA Gel Extraction Kit (Axygen Biosciences, Union City, CA, USA) and quantified with the Quanti FluorTM-ST (Promega, USA) according to the standard protocols. Sequencing was then performed on the Illumina MiSeq HiSeq platform (Illumina Inc., San Diego, CA). Operational taxonomic units (OTUs) were picked with a threshold of 97% sequence, and the identified taxonomy was aligned with the Silva database (Release 128). At a given taxonomic rank, 16S rRNA sequences was assigned using the RDP classifier (version 2.2).

### Metabolomics analysis on serum

After therapy, whole blood was collected from the orbital vein plexus, centrifuged for 15 min at 15,000 rpm for 20 min, and the serum sample was obtained for UPLC-Q-TOF/MS analysis(17). The separation was performed on a Hypesil Gold column (100×2.1 mm, 1.9µm) at a flow rate of 0.2mL/min. The mobile phase consisted of 0.1% formic acid in water as solvent A and acetonitrile as solvent B, and the solvent gradient was set as follows: 0–3 min, 5%–45% B; 3–13 min, 40%–95% B; 13–14 min, 95% B; 14–15 min, 95%–5% B.

The scanning of mass spectrometry was performed for the positive and negative ionization modes, respectively. The optimal conditions were set as follows: Positive ion mode: ion source temperature, 100 °C; capillary voltage, 2.5 kV; cone voltage, 24 V; desolvation gas, 800 L/h, cone gas flow, 50 L/h. Negative ion mode: ion source temperature, 100 °C; capillary voltage, 2.5 kV; cone voltage, 25 V; desolvation gas, 600 L/h, cone gas flow, 10 L/h. The production MS scan range was from 50 to 1500 Da, and TOF-MS scanning range was from 100 to 2000 Da.

The UPLC-Q/TOF-MS original data were processed using the MarkViewTM software. Principal components analysis (PCA), orthogonal partial least squares-discriminant analysis (OPLS-DA), and Partial least squares discriminant analysis (PLS-DA) were performed at metaX to cluster the sample plots across different groups. The metabolites with variable importance (VIP) > 1 and *p*-value< 0.05 were selected as the differential metabolites. The HMDB Database (http://www.hmdb.ca/), METLIN Database (http://metlin.scripps.edu/), MassBank Database (http://www.massbank.jp/) and KEGG Database (http://www.genome.jp/kegg/) were utilized to identify the potential biomarkers and metabolic pathways.

### Statistical analysis

Statistical significance was analyzed with SPSS 22.0 (SPSS Inc., Chicago, IL, USA). Quantitative data were presented as mean ± standard deviation (SD), and statistical significance was defined by *P*<0.05. The comparisons between two groups made using two-tailed unpaired Student’s t-tests, and the comparisons between multiple groups made using one-way analysis of variance (ANOVA). Pearson’s correlation coefficient was established to show the association between the altered gut microbiota and disturbed serum metabolites.

## Results

### Anti-tumor efficacy of XJR *in vitro* and *vivo*

**Figure 1:**
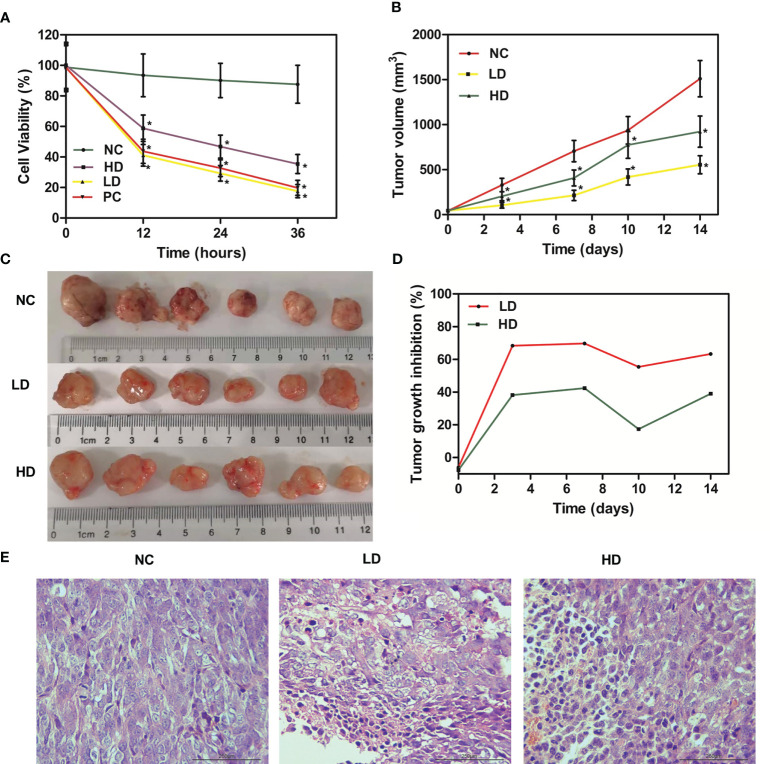
Anti-tumor effect of XJR *in vitro* and *in vivo*. **(A)** Cell viability was determined by MTT assay. **(B)** Tumor volume changes of tumor-bearing mice in different treatment groups. **(C)** Tumor photographs of different treatment groups after 14 days. **(D)** Tumor growth inhibition rate (TGI) of mice in different treatment groups. **(E)** H&E staining of tumor samples. NC, control group; LD, low-dose group of XJR; HD, high-dose group of XJR; PC, positive group; XJR, Xiaoai Jiedu Recipe. H&E, hematoxylin and eosin; TGI, tumor growth inhibition rate. *vs. NC, p < 0.05.

MTT assay was utilized to assess the effect of XJR on proliferation ability of CRC cells. We found that XJR significantly suppressed DLD-1 cells proliferation compared with the NC group, and the inhibitory effect of low-dose of XJR was stronger than that of high-dose of XJR (**Figure 1A**).

In this study, we also investigated the *in vivo* effects of XJR therapy on the growth of CRC with HCT116 tumor-bearing mice. Compared with the NC group, tumor progression was dramatically inhibited by LD therapy, and the HD group suppressed tumor growth to a certain degree. The relative tumor volume of LD and HD groups were smaller than that of the NC group, and the tumor volume of LD group was also smaller than that of HD group (**Figures 1B, C**). During the treatment phase, we also conducted the TGI score to evaluate the efficacy. The result showed that the TGI of the LD and HD groups were significant higher than that of the NC group, and the TGI of the LD group was more significant, consistent with the results of MTT and tumor volume (**Figure 1D**).

Meanwhile, we performed H&E staining on tumor samples to better demonstrate the anti-tumor effect of XJR *in vivo*. According to the histopathology changes, we found that there was moderate damage to the tumor tissue in the HD group, and the structural damage of the tumor tissue in the LD group was more apparent and extensive, such as irregular widening of the intercellular space and severe necrosis (**Figure 1E**). In conclusion, these results showed that XJR can inhibit tumor growth of CRC *in vitro* and *vivo*, and the LD therapy had the stronger inhibitory effect on tumor progression. Therefore, we chose the low dose of XJR for the following experiments.

### Gut microbiota analysis

**Figure 2:**
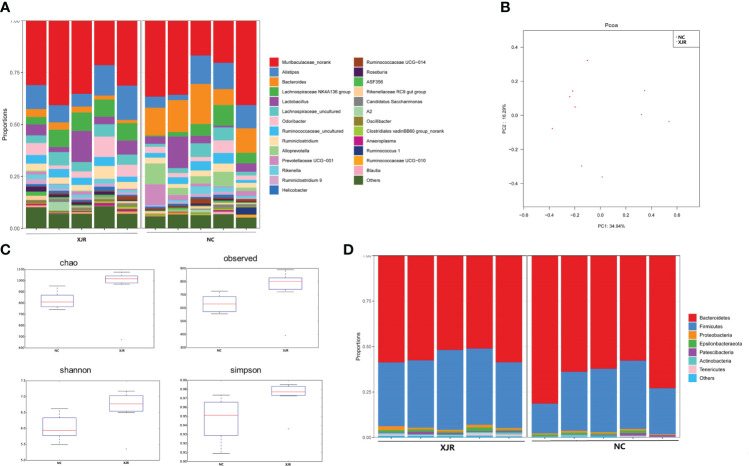
XJR modulated the gut microbiome composition. **(A)** Bar plot of the relative composition of gut microbiome at the genus level in the NC and XJR groups. **(B)** PCoA score plots in different groups. **(C)**Alpha diversity of the gut microbiome between the NC and XJR groups based on Chao, Observed, Shannon, and Simpson indices. **(D)** Relative gut microbiota abundance at the phylum level in the NC and XJR groups. NC, control group; XJR, Xiaoai Jiedu Recipe.

In order to explore whether the anti-tumor efficacy of XJR was associated with fecal microbiota, we analyzed the altered fecal microbiota composition profiles after XJR treatment by the 16S rDNA sequencing method. We assessed the phylogenetic composition of the intestinal flora in the NC group and XJR treatment group, and found the relative diversity of communities at the genus level in the different groups (**Figure 2A**). Principal coordinate analysis (PCoA) was preformed to compare the microbial community structure. PCoA analysis revealed the community composition of the NC group was far from that of the XJR treatment group, indicating that the flora composition had significant changes after XJR treatment (**Figure 2B**). Based on Chao, Observed, Shannon, and Simpson indices, there was no significant difference observed in the alpha diversity of the intestinal flora between the NC group and the XJR group (**Figure 2C**). *Firmicutes* and *Bacteroidetes* accounted for more than 90% at the phylum level (**Figure 2D**). Compared with the NC group, the proportion of *Bacteroidetes* in the XJR group decreased to 56.6%, and that of *Firmicutes* increased to 38.8%.

**Figure 3:**
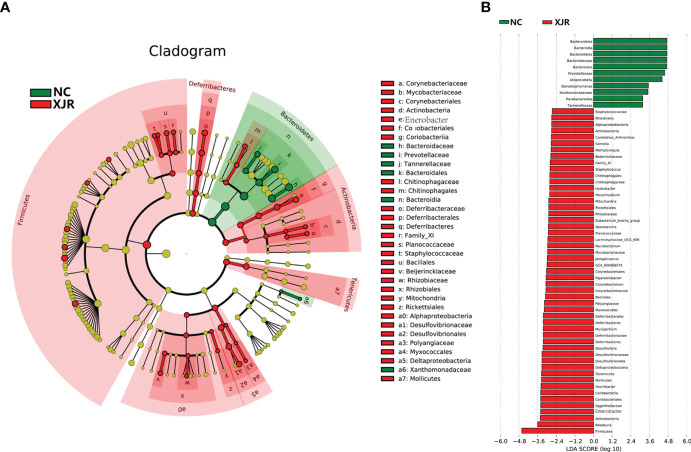
LEfSe analysis. **(A)** LDA scores of different abundant taxa in the faecal microbiomes from the NC and XJR groups. **(B)** Taxonomic cladogram from LEfSe. NC, control group; XJR, Xiaoai Jiedu Recipe.

To further identify the dominant altered fecal microbiota composition profiles after XJR treatment, we performed the high-dimensional class comparison through linear discriminant analysis effect size (LEfSe) analysis. The results again demonstrated there were differences in the abundance of bacteria between the different groups, with *Bacteroidales*, *Bacteroidia, Bacteroidetes*, *Bacteroides*, and *Prevotellaceae* enriched in the NC group and *Firmicutes*, *Roseburia*, *Actinobacteria*, *Enterobacter*, and *Deferribacteres* enriched in the XJR group (**Figures 3A, B**).

### Serum metabolomics

**Figure 4:**
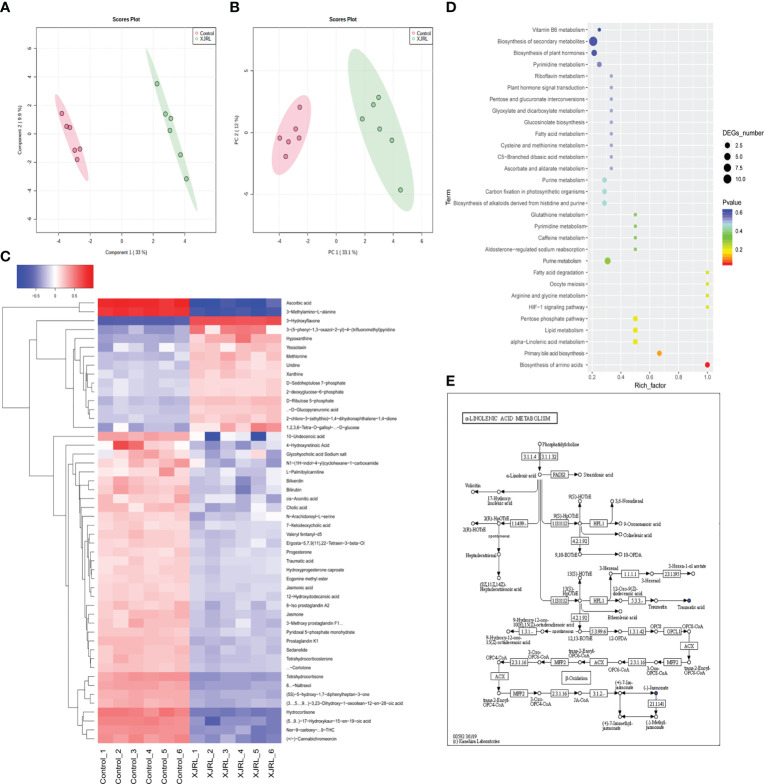
XJR altered serum metabolic profiles. **(A)** PCA plots and **(B)** OPLS-DA plots of the serum metabolic from NC and XJR groups. **(C)** Heat map showed normalised relative abundances of metabolites in the XJR group were significantly changed from those in the NC group. **(D)** Pathway enrichment of differential metabolites in the XJR group compared with the NC group. **(E)** Overview of alpha−linolenic acid metabolism with MetPA. NC, control group; XJR, Xiaoai Jiedu Recipe.

The altered serum metabolic profiles after XJR treatment was acquired by UPLC-MS. PCA results showed that the QC samples were closely clustered in the score plots, indicating that the analytical methods had good data quality and reproducibility (**Figure 4A**). Moreover, OPLS-DA model showed significant differences in the distribution of serum metabolic profile between the NC group and the XJR-treatment group (**Figure 4B**), suggesting that XJR led to significant biochemical changes. Identify metabolites involved in the clustering according to variable importance (VIP) values >1.0 and *p*-values<0.05, and these metabolites were selected for further identification. The HMDB Database (http://www.hmdb.ca/), METLIN Database (http://metlin.scripps.edu/), MassBank Database (http://www.massbank.jp/) and KEGG Database (http://www.genome.jp/kegg/) were utilized to identify the potential biomarkers.

Finally, 50 serum metabolites with different abundances were identified. The heat map showed that the serum metabolites in the XJR-treatment group were well separated from those in the NC group, and the variation tendencies of these metabolites were also described in the heat map (**Figure 4C**). Thirteen serum metabolites (**Table 1**) were significantly increased and thirty-seven (**Table 2**) were significantly decreased after treatment of XJR compared with control.

**Table 1:** Identified up-regulated metabolites in serum after treatment with XJR.

Compound_ID	Name	Formula	Molecular Weight	RT [min]	m/z	log2(XJR/NC)	P-value	VIP
Com_4158_neg	3-Hydroxyflavone	C_15_ H_10_ O_3_	238.0626	8.696	237.05536	4.42	4.66E-17	6.4356
Com_1884_pos	3-(5-phenyl-1,3-oxazol-2-yl)-4-(trifluoromethyl)pyridine	C_15_H_9_F_3_N_2_O	290.06253	1.393	291.06979	2.45	3.66E-05	3.3712
Com_384_pos	Hypoxanthine	C_5_H_4_N_4_O	136.03874	3.064	137.04601	2.13	1.25E-07	3.0378
Com_11633_pos	1,2,3,6-Tetra-O-galloyl-┐-D-glucose	C_34_H_28_O_22_	770.10082	10.015	771.10809	1.7	0.00026952	2.4559
Com_284_neg	D-Ribulose 5-phosphate	C_5_H_11_OP	230.0187	1.159	229.01151	1.55	4.42E-10	2.2655
Com_320_pos	Methionine	C_5_H_11_ NO_2_S	149.05132	1.88	150.05853	1.55	2.35E-07	2.2479
Com_1799_pos	2-chloro-3-(ethylthio)-1,4-dihydronaphthalene-1,4-dione	C_12_H_9_ClO_2_S	252.0011	1.397	253.00844	1.34	1.19E-09	1.946
Com_267_neg	β-D-Glucopyranuronic acid	C_6_H_10_O_7_	194.04208	1.16	193.03491	1.29	1.91E-10	1.8873
Com_6317_pos	Yessotoxin	C_55_H_82_O_21_S_2_	1124.45176	8.488	1125.45959	1.23	0.00012426	1.8563
Com_1072_neg	Uridine	C_9_H_12_N_2_O_6_	244.06905	2.136	243.0619	1.22	2.50E-07	1.7827
Com_312_pos	Xanthine	C_5_H_4_N_4_O_2_	152.03365	2.003	153.04091	1.14	3.89E-08	1.6664
Com_916_neg	2-deoxyglucose-6-phosphate	C_6_ H_13_O_8_P	244.03412	1.152	243.02672	1.03	1.04E-08	1.505
Com_920_neg	D-Sedoheptulose 7-phosphate	C_7_H_15_O_10_P	290.03972	1.151	289.0325	1.01	6.85E-09	1.4722

**Table 2:** Identified down-regulation metabolites in serum after treatment with XJR.

Compound_ID	Name	Formula	Molecular Weight	RT [min]	m/z	log2(XJR/NC)	P-value	VIP
Com_1005_neg	Ascorbic acid	C_6_H_8_O_6_	176.03181	1.205	175.02458	-5.51	4.38E-10	8.1635
Com_2226_neg	3-Methylamino-L-alanine	C_4_H_10_N_2_O_2_	118.07813	8.966	117.07087	-5.3	2.11E-13	7.7275
Com_4936_pos	Hydrocortisone	C_21_H_30_O_5_	362.20912	10.83	363.21649	-3.2	6.71E-13	4.649
Com_1098_neg	(5,9)-17-Hydroxykaur-15-en-19-oic acid	C_20_H_30_O_3_	364.22461	11.567	363.21725	-2.96	1.88E-09	4.3485
Com_5902_pos	Nor-9-carboxy-┘9-THC	C_21_H_28_O_4_	344.1986	10.832	345.20605	-2.74	7.50E-11	4.0123
Com_3800_pos	(+/-)-Cannabichromeorcin	C_17_H_22_O_2_	258.16506	12.204	259.17136	-2.71	9.70E-10	3.9576
Com_2297_pos	Tetrahydrocortisone	C_21_H_32_O_5_	346.21415	12.209	347.22141	-2.51	8.95E-13	3.6513
Com_4746_pos	6┐-Naltrexol	C_20_H_25_NO_4_	343.18144	12.959	344.18857	-2.3	5.51E-13	3.3442
Com_7834_pos	4-Hydroxyretinoic Acid	C_20_H_28_O_3_	316.20334	10.496	317.21057	-2.02	0.0017929	2.6141
Com_730_pos	(5S)-5-hydroxy-1,7-diphenylheptan-3-one	C_19_H_22_O_2_	282.16495	12.235	283.17236	-1.99	6.09E-14	2.9013
Com_2857_pos	(3,5,9)-3,23-Dihydroxy-1-oxoolean-12-en-28-oic acid	C_30_H_46_O_5_	486.33493	13.105	487.34222	-1.9	4.17E-11	2.7722
Com_953_neg	10-Undecenoic acid	C_11_H_20_O_2_	184.14598	12.441	183.13875	-1.74	0.0046333	3.222
Com_5034_neg	Prostaglandin K1	C_20_H_32_O_5_	334.214	11.352	333.20682	-1.6	9.08E-10	2.3364
Com_2319_pos	Bilirubin	C_33_H_36_N_4_O_6_	584.26344	15.674	585.27039	-1.59	6.90E-06	2.3585
Com_1955_pos	Sedanolide	C_12_H_18_O_2_	194.13094	13.03	195.13831	-1.54	1.27E-09	2.2557
Com_1680_pos	Tetrahydrocorticosterone	C_21_H_34_O_4_	367.27195	12.44	368.27899	-1.53	7.42E-10	2.2289
Com_1708_pos	Jasmone	C_11_H_16_O	164.12026	13.594	165.12756	-1.5	7.84E-09	2.1808
Com_5743_pos	3-Methoxy prostaglandin F1┐	C_21_H_38_O_6_	346.27162	13.778	347.27872	-1.48	3.33E-07	2.1713
Com_1427_pos	┐-Cortolone	C_21_H_34_O_5_	348.22988	11.497	349.23715	-1.44	1.74E-11	2.1012
Com_2561_neg	Cholic acid	C_24_H_40_O_5_	408.28704	11.625	407.27991	-1.44	1.18E-05	2.1434
Com_9615_pos	N1-(1H-indol-4-yl)cyclohexane-1-carboxamide	C_15_H_18_N_2_O	280.09694	14.16	281.10434	-1.43	0.00021423	2.1856
Com_5966_neg	8-Iso prostaglandin A2	C_20_H_30_O_4_	334.21402	10.489	333.20676	-1.37	5.31E-07	2.0217
Com_2598_pos	Biliverdin	C_33_H_34_N_4_O_6_	582.24771	15.671	583.25513	-1.36	3.14E-05	2.0276
Com_4412_neg	Pyridoxal 5-phosphate monohydrate	C_8_H_12_NO_7_P	265.03606	7.671	264.02863	-1.36	1.44E-09	1.992
Com_4554_neg	cis-Aconitic acid	C_6_H_6_O^6^	174.01655	1.5	173.00958	-1.28	4.26E-05	1.8933
Com_979_pos	Valeryl fentanyl-d5	C_24_H_27[2]_H_5_N_2_O	369.28761	12.786	370.29507	-1.27	1.37E-08	1.8641
Com_2295_neg	12-Hydroxydodecanoic acid	C_12_H_24_O_3_	216.17218	12.525	215.16496	-1.16	3.59E-10	1.6995
Com_5676_pos	Ergosta-5,7,9(11),22-Tetraen-3-beta-Ol	C_28_H_42_O	394.32333	13.567	395.33075	-1.14	1.42E-07	1.6818
Com_1173_pos	Ecgonine methyl ester	C_10_H_17_NO_3_	199.12111	10.759	200.12846	-1.11	2.02E-10	1.6137
Com_6024_pos	N-Arachidonoyl-L-serine	C_23_H_37_NO_4_	391.27178	12.457	392.27921	-1.1	9.07E-05	1.678
Com_820_neg	Jasmonic acid	C_12_H_18_O_3_	210.12528	12.049	209.11801	-1.1	2.16E-11	1.6082
Com_2031_neg	Glycohyocholic acid Sodium salt	C_26_H_42_NNaO^6^	487.29247	14.138	486.28516	-1.08	0.0075724	1.6385
Com_2962_pos	Hydroxyprogesterone caproate	C_27_H_40_O_4_	428.29253	13.324	429.29971	-1.08	2.48E-08	1.5725
Com_7398_pos	L-Palmitoylcarnitine	C_23_H_45_NO_4_	399.33412	13.741	400.3414	-1.08	0.00037143	1.5228
Com_4342_pos	Progesterone	C_21_H_30_O_2_	314.22432	13.183	315.2316	-1.05	2.32E-08	1.5384
Com_1136_pos	Traumatic acid	C_12_H_20_O_4_	228.13631	13.075	229.14378	-1	4.71E-08	1.462
Com_8893_neg	7-Ketodeoxycholic acid	C_24_H_38_O_5_	406.27131	12.054	405.26404	-1	3.00E-07	1.4677

Further pathway enrichment and network possibly affected by XJR were searched *via* Metabo Analyst. It mainly involved 12 metabolic pathways, including the biosynthesis of amino acids, primary bile acid biosynthesis, alpha−linolenic acid metabolism, ascorbic acid pathway, pentose phosphate pathway, HIF−1 signaling pathway, arginine and glycine metabolism, fatty acid degradation, purine metabolism, lipid metabolism, pyrimidine metabolism, and glutathione metabolism (**Figure 4D**). An overview of correlation analysis of alpha−linolenic acid metabolism according to Metabo was shown in **Figure 4E**.

### Associations between the gut microbiota and serum metabolic phenotype

**Figure 5:**
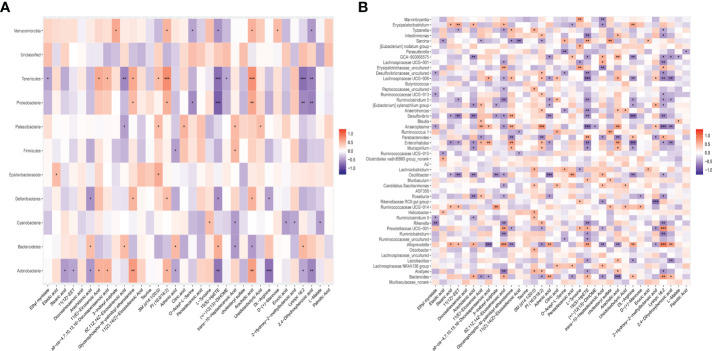
The close correlations between the relative abundance of gut microbiota and potential biomarkers. **(A)** The close correlations between the relative abundance of the main phylum and **(B)** genus in the gut microbiota and the potential serum biomarkers differently expressed between the NC and XJR group. NC, control group; XJR, Xiaoai Jiedu Recipe. *, p < 0.05; **, p< 0.01; ***, p < 0.001.

To investigate the associations between the altered gut microbiota and disturbed serum metabolites, we calculated the correlation matrixe based on Pearson’s correlation coefficients. We found a strong correlation between some bacteria and metabolites (r > 0.5 or r < –0.5, p<0.05). At the phylum level, there was a significant negative correlation between the protective bacteria such as *Actinobacteria*, *Firmicutes*, and *Deferribacteres* and *Arachidonic acid*, *Adrenic acid*, *15(S)*−*HpETE*, *DL−Arginine* and *Lysopc 18:2*. However, there was a significant positive correlation between the aggressive bacteria such as *Bacteroidetes* and *Arachidonic acid*, *Adrenic acid*, *15(S)−HpETE*, *DL−Arginine* and *Lysopc 18:2* (**Figure 5A**). At the genus level, *Roseburia* and *Enterorhabdus* were negatively correlated with *Arachidonic acid*, *Adrenic acid*, *15(S)*−*HpETE*, *DL−Arginine* and *Lysopc 18:2*. *Bacteroides Prevotellaceae* and *UCG−001* were positively correlated with *Arachidonic acid*, *Adrenic acid*, *15(S)−HpETE*, *DL−Arginine* and *Lysopc 18:2* (**Figure 5B**). These results indicated that the altered intestinal flora was closely related to the arachidonic acid metabolism, and these two factors interacted to modulate inflammation. XJR may play a role in inhibiting CRC by fostering the growth of beneficial gut microbiota, inhibiting the growth of harmful gut microbiota, regulating the intestinal microecology balance and restoring inflammation.

## Discussion

CRC is one of the most common cancers of the digestive system with a high mortality rate, and causing serious health issues worldwide. Despite advances in treatment strategies, the prognosis for CRC remains unsatisfactory (18). As a classical anti-tumor prescription, XJR is composed of *Hedyotis diffusa*, *Radix pseudostellariae*, *Akebia trifoliata Koidz*, *Radix ophiopogonis*, *Bombyx batryticatus*, *Cremastra appendiculata*, and *Centipede* in a specific proportion. Although XJR has been clinically proven to prolong the survival and improve the quality of life of patients with various malignant tumors, its mechanism of action has not been clarified. In the study, we investigated the anti-CRC activity of XJR *in vivo* and *vitro*. The results indicated that XJR could inhibit tumor growth of CRC *in vitro* and *vivo*, and the low-dose XJR had the stronger inhibitory effect on tumor progression rather than the high-dose XJR. The complex mechanisms involved need to be further explored.

As a multifactorial disease, increasing researches had shown that intestinal flora played a decisive role in the occurrence and development of CRC, which may provide new treatment strategy of CRC (19). Therefore, we explored the regulatory effect of XJR on intestinal microflora in tumor-bearing mice with 16S rRNA gene sequencing. Compared with pre-treatment of XJR, the abundance of *Bacteroidetes, Bacteroides*, and *Prevotellaceae* decreased, while the abundance of *Firmicutes*, *Roseburia*, and *Actinobacteria* increased post-treatment. As opportunistic pathogens, the contents of *Bacteroidetes, Bacteroides*,and *Prevotellaceae* in CRC patients were higher than in healthy volunteers, which could be used as non-invasive markers for detection of CRC (20). Yuan et al. also found that at the phylum level, the abundance of *Firmicutes* and *Actinobacteria* was higher in healthy samples than in CRC patients, suggesting that they were probably “favourable”(20). Wang et al. found that the abundance of *Roseburia* was lower in CRC patients compared to normal people (21). In our study, *Roseburia* was significantly enriched after the treatment of XJR. Therefore, consistent with previous studies, we considered *Roseburia* to be the “beneficial bacteria”. As expected, the results demonstrated that XJR might effectively intervene in the progression of CRC by altering the composition profiles of fecal microbiota.

Metabolomics focuses on investigating the composition and change of endogenous metabolites in the biological specimen, which could reflect the impact of internal and external factors on their overall metabolism (22). In this study, there were 12 metabolic pathways possibly affected by XJR, among which the primary bile acid biosynthesis, alpha−linolenic acid metabolism, and ascorbic acid pathway had particularly attracted our attention. Liu et al. found the imbalance of bile acid metabolism can promote the development of CRC (23). Blood level of alpha−linolenic was inversely associated with the risk of CRC, which suggested that alpha−linolenic could be biomarkers for monitoring efficacy in patients with CRC (24). It has been reported that ascorbic acid could promote tumor regression, improved tolerability and side effects in patients with advanced CRC(25). Hence, we speculated that XJR play a role in inhibiting CRC by regulating the pathways of primary bile acid, alpha−linolenic acid, and ascorbic acid metabolism. We found that thirteen serum metabolites were more abundant while another thirty-seven serum metabolites were less abundant in the XJR group compared with the control group. In this study, the level of prostaglandin K1 was significantly reduced after the administration of XJR. Prostaglandins are inflammatory mediators generated from arachidonic acids under the action of cyclooxygenase-2 (COX-2), which are related to pathological processes, such as inflammation, the occurrence and progression of cancer, and cardiovascular disease(26). Previous studies have report that prostaglandin could promote CRC occurrence and progression, and inhibition of prostaglandin with COX-2 selective inhibitors or non-steroidal anti-inflammatory drugs (NSAIDs) could prevention of CRC (27, 28). Therefore, based on the above metabolomics results, we speculated that XJR could interfere in the development of CRC by regulating these metabolites.

Accumulated data indicate that the intestinal microbiota disturbances related to changes in metabolic phenotypes have been used to explore the possible mechanisms of disease progression and drug therapy (29). Herein, we investigated the correlation between altered gut microbiota and disturbed serum metabolites using Pearson’s correlation analysis. Of particular interest, there was a significant negative correlation between the protective bacteria such as *Firmicutes*, *Roseburia*, and *Actinobacteria* and *Arachidonic acid*, *Adrenic acid*, *15(S)*−*HpETE*, *DL−Arginine* and *Lysopc 18:2*. The aggressive bacteria such as *Bacteroidetes*, *Bacteroides*, and *Prevotellaceae* were positively correlated with *Arachidonic acid*, *Adrenic acid*, *15(S)−HpETE*, *DL−Arginine* and *Lysopc 18:2*. These results indicated that the altered intestinal flora was closely related to the arachidonic acid metabolism, and these two factors interacted to modulate inflammation(30). XJR may play a role in inhibiting CRC by fostering the growth of beneficial gut microbiota, inhibiting the growth of harmful gut microbiota, regulating the intestinal microecology balance and restoring inflammation. However, further research is needed to confirm this hypothesis.

In this study, XJR displayed significant anti-tumor effects without obvious side effects, suggesting it is a promising treatment for CRC. We also found that XJR significantly altered not only the fecal microbiota composition but also serum metabolic profiles. Furthermore, correlation analysis revealed that the altered gut microbiota had significant correlations with disturbed serum metabolites. However, we temporarily have no evidence to show that there is a direct causal connection between the ability of XJR to regulate intestinal flora, alter serum metabolic profile, and its ability to ameliorate CRC, which will be the focus of our next study. Overall, the regulation of gut microbiota and related metabolites may be potential breakthrough point to elucidate the mechanism of XJR in the treatment of the CRC. The strategy employed would provide theoretical basis for clinical application of TCM.

## Data availability statement

The datasets presented in this study can be found in online repositories. The names of the repository/repositories and accession number(s) can be found in the article/supplementary material.

## Ethics statement

The animal study was reviewed and approved by Nanjing University of Chinese Medicine.

## Author contributions

Design of experiment: HGZ; paper drafting: WQ; *in vitro* experiment: XH; establishment of mouse model and *in vivo* experiment: HC and XH; data analysis: WQ and XH; paper correction: WQ, HLZ and ZW. All authors contributed to the article and approved the submitted version.
